# Supplementary material for: Transcriptome Analysis Reveals Vimentin-Induced Disruption of Cell–Cell Associations Augments Breast Cancer Cell Migration
Source: Cells. 2022 Dec 13;11(24):4035. doi: 10.3390/cells11244035 (PMC9776984; doi:10.3390/cells11244035)
Supplement: Supplementary file 1 [file cells-11-04035-s001.zip › DEGs and enrichment analysis.zip!/DEGs and enrichment analysis/WT_VIMvsVector GO enrichment/GSEA/WT_VIM/SCAFFOLD_PROTEIN_BINDING/SCAFFOLD_PROTEIN_BINDING(GO_0097110).html]

Details for gene set SCAFFOLD\_PROTEIN\_BINDING(GO:0097110)[GSEA]


|  || Dataset | fpkm.sample |
| Phenotype | sample.cls |
| Upregulated in class | WT\_VIM |
| GeneSet | SCAFFOLD\_PROTEIN\_BINDING(GO:0097110) |
| Enrichment Score (ES) | 0.9277116 |
| Normalized Enrichment Score (NES) | 1.4851592 |
| Nominal p-value | 0.0 |
| FDR q-value | 0.1629998 |
| FWER p-Value | 0.163 |
Table: GSEA Results Summary

  

Fig 1: Enrichment plot: SCAFFOLD\_PROTEIN\_BINDING(GO:0097110)
  
Profile of the Running ES Score & Positions of GeneSet Members on the Rank Ordered List

  

| PROBE | DESCRIPTION   (from dataset) | GENE SYMBOL | GENE\_TITLE | RANK IN GENE LIST | RANK METRIC SCORE | RUNNING ES | CORE ENRICHMENT || 1 | ENSG00000089250 | NOS1 |  |  | 1293 | Infinity | -0.0219 | Yes |
| 2 | ENSG00000026025 | VIM |  |  | 2388 | 1709.539 | 0.9277 | Yes |
| 3 | ENSG00000055118 | KCNH2 |  |  | 3834 | 3.233 | 0.9051 | No |
| 4 | ENSG00000122966 | CIT |  |  | 4506 | 2.645 | 0.8952 | No |
| 5 | ENSG00000108953 | YWHAE |  |  | 5140 | 2.324 | 0.8858 | No |
| 6 | ENSG00000126934 | MAP2K2 |  |  | 6037 | 2.023 | 0.8718 | No |
| 7 | ENSG00000070814 | TCOF1 |  |  | 6098 | 2.004 | 0.8719 | No |
| 8 | ENSG00000102882 | MAPK3 |  |  | 6737 | 1.867 | 0.8622 | No |
| 9 | ENSG00000132535 | DLG4 |  |  | 7398 | 1.736 | 0.8520 | No |
| 10 | ENSG00000175866 | BAIAP2 |  |  | 7526 | 1.711 | 0.8508 | No |
| 11 | ENSG00000134569 | LRP4 |  |  | 7635 | 1.689 | 0.8499 | No |
| 12 | ENSG00000105401 | CDC37 |  |  | 7777 | 1.661 | 0.8485 | No |
| 13 | ENSG00000251322 | SHANK3 |  |  | 7906 | 1.640 | 0.8473 | No |
| 14 | ENSG00000108312 | UBTF |  |  | 7949 | 1.631 | 0.8475 | No |
| 15 | ENSG00000167193 | CRK |  |  | 8813 | 1.506 | 0.8337 | No |
| 16 | ENSG00000186081 | KRT5 |  |  | 8964 | 1.487 | 0.8320 | No |
| 17 | ENSG00000170421 | KRT8 |  |  | 9203 | 1.452 | 0.8288 | No |
| 18 | ENSG00000111057 | KRT18 |  |  | 10205 | 1.334 | 0.8126 | No |
| 19 | ENSG00000197122 | SRC |  |  | 11158 | 1.251 | 0.7972 | No |
| 20 | ENSG00000161681 | SHANK1 |  |  | 11845 | 1.196 | 0.7863 | No |
| 21 | ENSG00000169032 | MAP2K1 |  |  | 11855 | 1.195 | 0.7868 | No |
| 22 | ENSG00000116288 | PARK7 |  |  | 12017 | 1.180 | 0.7847 | No |
| 23 | ENSG00000110218 | PANX1 |  |  | 12148 | 1.169 | 0.7832 | No |
| 24 | ENSG00000171346 | KRT15 |  |  | 12263 | 1.159 | 0.7819 | No |
| 25 | ENSG00000058668 | ATP2B4 |  |  | 13275 | 1.080 | 0.7654 | No |
| 26 | ENSG00000113448 | PDE4D |  |  | 13773 | 1.044 | 0.7576 | No |
| 27 | ENSG00000006283 | CACNA1G |  |  | 13803 | 1.041 | 0.7577 | No |
| 28 | ENSG00000088986 | DYNLL1 |  |  | 14027 | 1.023 | 0.7545 | No |
| 29 | ENSG00000196557 | CACNA1H |  |  | 15033 | 0.968 | 0.7381 | No |
| 30 | ENSG00000071051 | NCK2 |  |  | 15594 | 0.933 | 0.7291 | No |
| 31 | ENSG00000135341 | MAP3K7 |  |  | 17194 | 0.845 | 0.7025 | No |
| 32 | ENSG00000105971 | CAV2 |  |  | 17639 | 0.820 | 0.6955 | No |
| 33 | ENSG00000213341 | CHUK |  |  | 17817 | 0.810 | 0.6929 | No |
| 34 | ENSG00000264364 | DYNLL2 |  |  | 18065 | 0.796 | 0.6892 | No |
| 35 | ENSG00000080824 | HSP90AA1 |  |  | 18749 | 0.757 | 0.6781 | No |
| 36 | ENSG00000104365 | IKBKB |  |  | 18846 | 0.751 | 0.6769 | No |
| 37 | ENSG00000064012 | CASP8 |  |  | 18979 | 0.744 | 0.6750 | No |
| 38 | ENSG00000156531 | PHF6 |  |  | 19084 | 0.737 | 0.6737 | No |
| 39 | ENSG00000096696 | DSP |  |  | 19375 | 0.721 | 0.6692 | No |
| 40 | ENSG00000169860 | P2RY1 |  |  | 19518 | 0.714 | 0.6672 | No |
| 41 | ENSG00000054523 | KIF1B |  |  | 20255 | 0.673 | 0.6551 | No |
| 42 | ENSG00000053918 | KCNQ1 |  |  | 21187 | 0.626 | 0.6397 | No |
| 43 | ENSG00000135679 | MDM2 |  |  | 21791 | 0.590 | 0.6299 | No |
| 44 | ENSG00000179841 | AKAP5 |  |  | 21885 | 0.583 | 0.6286 | No |
| 45 | ENSG00000123104 | ITPR2 |  |  | 23073 | 0.501 | 0.6088 | No |
| 46 | ENSG00000119878 | CRIPT |  |  | 23477 | 0.480 | 0.6022 | No |
| 47 | ENSG00000183873 | SCN5A |  |  | 23582 | 0.472 | 0.6008 | No |
| 48 | ENSG00000196338 | NLGN3 |  |  | 24384 | 0.409 | 0.5874 | No |
| 49 | ENSG00000164867 | NOS3 |  |  | 24772 | 0.375 | 0.5811 | No |
| 50 | ENSG00000173175 | ADCY5 |  |  | 24838 | 0.369 | 0.5802 | No |
| 51 | ENSG00000152661 | GJA1 |  |  | 25762 | 0.276 | 0.5647 | No |
| 52 | ENSG00000095970 | TREM2 |  |  | 27596 | 0.000 | 0.5337 | No |
| 53 | ENSG00000152208 | GRID2 |  |  | 33525 | NaN | 0.4334 | No |
| 54 | ENSG00000146938 | NLGN4X |  |  | 36477 | NaN | 0.3834 | No |
| 55 | ENSG00000124731 | TREM1 |  |  | 40121 | NaN | 0.3218 | No |
| 56 | ENSG00000169760 | NLGN1 |  |  | 46633 | NaN | 0.2116 | No |
| 57 | ENSG00000130037 | KCNA5 |  |  | 47042 | NaN | 0.2047 | No |
| 58 | ENSG00000198719 | DLL1 |  |  | 56017 | NaN | 0.0528 | No |
| 59 | ENSG00000165246 | NLGN4Y |  |  | 58388 | NaN | 0.0126 | No |
Table: GSEA details
[plain text format]

  

Fig 2: SCAFFOLD\_PROTEIN\_BINDING(GO:0097110)
  
Blue-Pink O' Gram in the Space of the Analyzed GeneSet

  

Fig 3: SCAFFOLD\_PROTEIN\_BINDING(GO:0097110): Random ES distribution
  
Gene set null distribution of ES for
 **SCAFFOLD\_PROTEIN\_BINDING(GO:0097110)**
